# Mechanical Behavior of Thin Ceramic Laminates on Central Incisors

**DOI:** 10.3390/ma17225663

**Published:** 2024-11-20

**Authors:** Stephanie Soares Favero, Kelli Nunes Monteiro, Aline Rodrigues, Ketuly Marques Cestari, Carlos Alberto Jurado, Abdulaziz Alhotan, Paulo Francisco Cesar

**Affiliations:** 1 Department of Biomaterials and Oral Biology, School of Dentistry, University of São Paulo, São Paulo 05508-000, Brazilknmonteiro1@gmail.com (K.N.M.);; 2 Operative Dentistry Division, Department of General Dentistry, College of Dentistry, University of Tennessee Health Science Center, Memphis, TN 38163, USA; 3 Department of Dental Health, College of Applied Medical Sciences, King Saud University, P.O. Box 10219, Riyadh 12372, Saudi Arabia

**Keywords:** lithium disilicate, feldspathic porcelain, computer-aided design, fracture load, thin ceramic laminates

## Abstract

Restorative dentistry often uses ceramic laminate veneers for aesthetic anterior teeth restorations due to their natural appearance and minimal invasiveness. However, the understanding of their clinical performance and how ceramic microstructure and processing affect longevity is limited. *Objective:* This study aimed to address this gap by determining the mechanical behavior, fracture load, and failure modes of CAD-CAM processed laminate veneers made of either lithium-disilicate-based glass ceramic (IPS e.max CAD) or feldspathic porcelain (Vita Mark II). It also aimed to develop a mechanical cycling methodology capable of determining the lifetime and failure modes of thin ceramic laminate veneers. *Materials and Methods:* Eighteen human maxillary central incisors were used to create the specimens. Minimal enamel preparation was required to ensure the proper adaptation of the thin ceramic laminates. Ceramic laminates made from lithium disilicate and feldspathic porcelain (Vita Mark II) were produced via CAD-CAM, with the final thicknesses less than 0.5 mm, then cemented with resin cement. *Results:* The mean fracture load for the glass ceramic was 431.8 ± 217.9 N, while for the porcelain, it was 454.4 ± 72.1 N. Failure modes differed considerably: porcelain showed more chipping, while lithium disilicate was associated with tooth structure failure. *Conclusion:* The material used did not significantly affect the fracture load of thin ceramic laminates in static tests. However, failure modes differed considerably. It was not possible to determine a set of mechanical cycling parameters that could establish the fatigue properties of thin ceramic laminates, as the maximum number of cycles reached was 536,818.

## 1. Introduction

Aesthetic restorations in anterior teeth are among the most challenging tasks in restorative dentistry. Ceramic materials, however, are widely considered to best mimic the appearance of human teeth [1,2]. Recent advances in ceramic processing methods have simplified the workflow in dental laboratories and allowed for greater control over the quality of ceramic restorations, improving their mechanical reliability [3]. As a result, the use of all-ceramic prostheses in restorative treatments has increased significantly worldwide [4].

Studies have explored the mechanical properties and clinical performance of laminate veneers under various conditions [5,6]. Modern CAD-CAM technology encompasses both subtractive and additive manufacturing processes. While traditional subtractive methods involve milling ceramic blocks, recent advances in additive manufacturing, such as selective laser sintering (SLS) and digital light processing (DLP), offer new possibilities for ceramic restoration fabrication. These additive processes can potentially reduce material waste and allow for more complex geometries compared to subtractive methods. However, current ceramic materials for additive manufacturing still face challenges regarding mechanical properties and aesthetic outcomes compared to established subtractive CAD-CAM materials like lithium disilicate and feldspathic porcelain. The continuous development of new ceramic materials and processing techniques may soon bridge this gap, potentially revolutionizing the fabrication of dental restorations [7].

Ceramic laminate veneers are becoming an increasingly popular solution for addressing aesthetic issues such as tooth morphology, color, size, and position [5]. These restorations are highly aesthetic and align with the minimally invasive philosophy by preserving significant amounts of dental structure [8]. In selected cases, minimal preparation is necessary [9]. Ceramic laminate veneers are categorized by thickness into three types: conventional laminates (0.5 mm to 1.0 mm), thin laminates (0.3 mm to 0.5 mm), and ultra-thin laminates (less than 0.3 mm) [10,11]. Ultra-thin laminates offer maximum preservation of tooth structure but present fabrication challenges. Thin laminates provide a balance between conservation and structural integrity, while conventional laminates offer superior durability but require more extensive tooth preparation [6,10,11]. Understanding these differences is crucial for optimizing restorative outcomes and advancing dental technologies.

Previous studies have shown that when enamel is preserved and veneers are bonded to this tissue, the longevity, and predictability of the restorations improve significantly compared to veneers bonded to dentin [12,13]. However, due to their thinness, ceramic laminate veneers are technically challenging to fabricate for both the dental technicians and clinicians. Any errors during production can significantly affect the longevity of the restoration in the oral cavity [14,15,16].

One critical factor influencing the longevity of laminate veneers is the ceramic processing method used [17]. CAD-CAM (computer-aided design computer-aided machining) processing involves machining ceramic blocks with carbide or diamond burs controlled by computer software [7]. This method reduces much of the manual labor by the dental technician. Moreover, since the ceramic blocks are produced under optimized conditions by manufacturers, they feature a highly controlled microstructure with low porosity and enhanced mechanical properties [4].

One of the most commonly used CAD-CAM processed ceramic materials for laminate veneers is lithium-disilicate-based glass-ceramic [18]. IPS e.max CAD is a well-known commercial example of this material, composed of a mixture of quartz powders, lithium dioxide, phosphorus oxide, aluminum oxide, potassium oxide, and other components. After crystallization, the ceramic contains approximately 70% lithium disilicate crystals by volume, with an average crystal size of 1.5 μm [19,20]. The flexural strength of IPS e.max CAD ranges from 360 to 400 MPa [21,22].

Feldspathic porcelains are often recommended for minimally invasive procedures that require high aesthetic outcomes, such as thin veneers [23]. CAD-CAM blocks made of feldspathic porcelain (Vita Mark II) exhibit flexural strength ranging from 130 to 160 MPa [24]. Their microstructure consists of leucite crystals, with an average size of 4 μm, dispersed within a glassy matrix, with crystalline content making up less than 20 vol% of the total volume [25].

A potential drawback of CAD-CAM processing is that hard machining can generate surface cracks along both the internal and external surfaces of the prosthetic piece. These cracks may develop into critical defects, which can slowly propagate under occlusal stress, leading to catastrophic failure when the critical stress threshold is reached [4]. The failure of ceramic dental prostheses is usually linked to microstructural defects such as pores and cracks, which can form during both the manufacturing process and mastication [26]. Micro-cracks are often undetectable to the naked eye but are among the most serious factors affecting the mechanical strength of ceramics [27].

Dental ceramics are inherently brittle materials, which means they cannot undergo significant plastic deformation before fracturing [28]. This lack of plasticity leads to high tensile stress concentrations around microstructural defects, accelerating crack propagation and causing catastrophic fractures [29]. Occlusal loading generates complex tensile, compressive, and shear stresses distributed throughout the cemented restoration [30].

Various in vitro methods have been used to replicate clinically relevant stresses in ceramic restorations to evaluate their fatigue behavior [31]. The design of the restoration and the type of load applied during these tests influence the mode of failure. Crack propagation in ceramic restorations is complex due to changes in the fracture plane, caused by the intricate geometry of the ceramic piece. Fractographic analysis helps to understand the crack propagation history and identify the origin of failure [32,33]. Further research is needed to better understand the variables affecting the fatigue behavior of thin veneers and to identify the types and locations of defects that lead to catastrophic fractures in these restorations.

Despite recent advances, there remains a limited understanding of how different ceramic microstructures and processing techniques influence the long-term performance and clinical outcomes of laminate veneers. This study seeks to address these gaps by evaluating the mechanical behavior, fracture load, and failure modes of CAD-CAM processed laminate veneers, specifically focusing on lithium-disilicate-based glass ceramic and feldspathic porcelain. Given the relatively recent adoption of this treatment and the various methodologies for fabricating thin ceramic laminate veneers, understanding how the ceramic microstructure and processing methods influence their longevity is crucial. By developing a mechanical cycling methodology, the study intends to provide deeper insights into the factors affecting the durability of thin ceramic veneers. Therefore, the purpose of this study was to determine the mechanical behavior, fracture load, and failure modes of CAD-CAM processed laminate veneers made of either lithium-disilicate-based glass ceramic or feldspathic porcelain. The study also aimed to develop a mechanical cycling methodology capable of determining the lifetime and failure modes of thin ceramic laminate veneers. The null hypotheses were as follows: (1) the microstructure of the ceramic material would not affect the fracture load of the thin ceramic laminate veneers, and (2) the microstructure of the ceramic material would not influence the failure mode of the thin ceramic laminate veneers.

## 2. Materials and Methods

**Figure 1:**
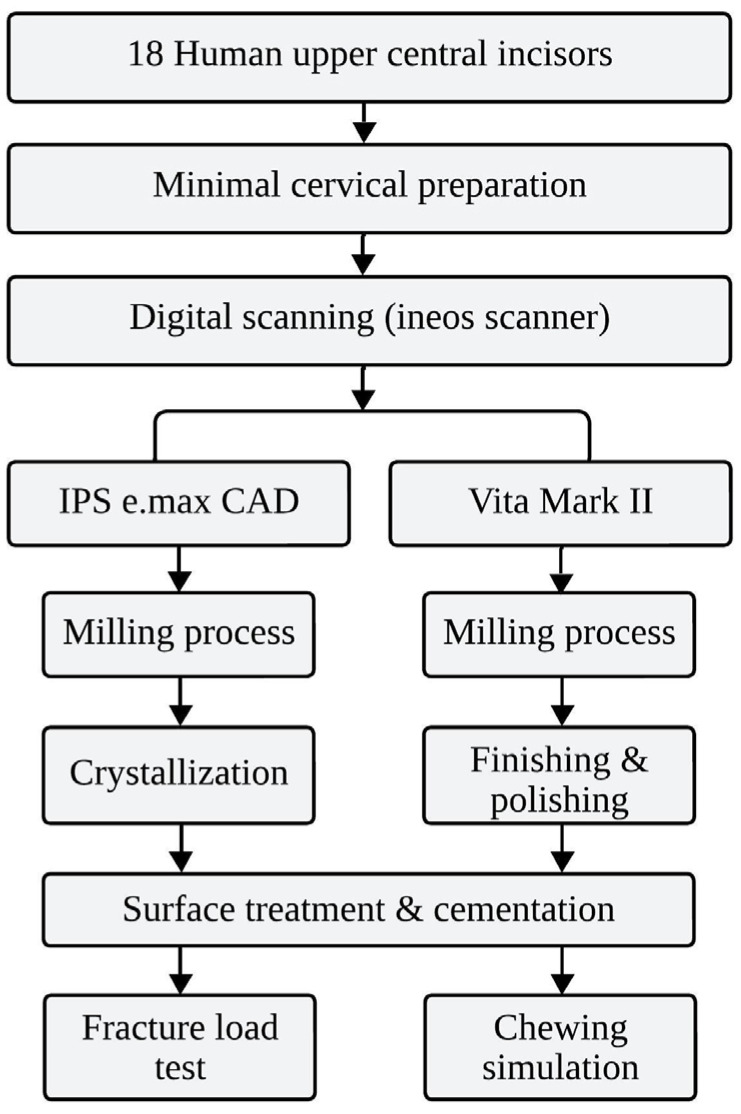
Experimental flowchart of the study design.

Eighteen human teeth, upper central incisors, were used to fabricate the specimens. The teeth were obtained from the tooth bank of the Dentistry School of the University of São Paulo with approval from the Research Ethics Committee (No. 44495315.9.0000.0075) of the same faculty. The selected teeth met the following criteria: (1) intact crown structure without previous restorations, caries, or visible cracks; (2) complete root formation; (3) similar crown dimensions (±0.5 mm in length and width); and (4) absence of developmental defects or structural anomalies. All teeth were examined under 2.5× magnification to ensure they met these criteria before inclusion in the study. The teeth were stored in water throughout the project as recommended by the tooth bank. The specimens for the current study were produced by luting thin ceramic laminates on eighteen upper central incisor human teeth. They were divided into test groups, as shown in Table 1. All testing procedures were conducted in a temperature-controlled laboratory maintained at 23 ± 1 °C with relative humidity of 50 ± 5%. Throughout the study, specimens were stored in distilled water at 37 °C as recommended by the tooth bank, with temperature monitored continuously using a calibrated digital thermometer. The experimental flowchart of this study is presented in Figure 1, illustrating the sequence from specimen preparation to testing procedures.

The ceramic materials, lithium disilicate (Ivoclar IPS e.max CAD) and feldspathic porcelain (Vita Mark II), were selected for their contrasting properties and clinical relevance, where lithium disilicate offers superior mechanical strength and feldspathic porcelain excels in aesthetics, thus making them suitable for comparing the performance of thin veneers in dental restorations.

Each tooth underwent a minimal cervical preparation for an adequate adaptation of the thin ceramic laminate. The proximal surfaces suffered minimal preparation to regularize the surface, since the objective was to simulate a minimally invasive restorative treatment. The incisal edge of the tooth was regularized to receive the thin ceramic laminate, simulating a clinical case in which the incisal edge was increased by 1.5 mm. After a minimally invasive preparation, the teeth were scanned using a digital scanner (Ineos, Sirona, Bensheim, Germany). After obtaining the image of the prepared tooth, a virtual image of the thin ceramic laminate was produced in the CEREC system with software version 5.1 (Sirona, Bensheim, Germany), simulating a thin restoration (thickness of 0.5 mm and 1.5 mm increase of the incisal edge).

The digital image was manipulated in the program, and the machining process commenced following the completion of the design. Lithium disilicate blocks IPS e.max CAD blocks (C14 with dimensions of 12 × 14 × 18 mm^3^) and porcelain blocks Vita Mark II (I14 with dimensions of 12 × 14 × 18 mm^3^) were used. At the end of the machining process, the thin IPS e.max CAD ceramic laminates were further crystallized in a ceramic furnace (Kerampress, Kota, Sao Paulo, Brazil). The crystallization process lasted 25 min at 850 °C, producing a controlled growth of lithium disilicate crystals. The thin laminates of both materials, with an initial thickness of 0.5 mm, were tested for adaptation on the corresponding tooth, and any necessary adjustments were made. The last step before cementation was the finishing and polishing step with specific rubbers for ceramic restorations (Exa Cerapol, Edenta, São Paulo, Brazil). The thickness of each ceramic laminate was measured using a digital caliper (Mitutoyo, Tokyo, Japan) at three points: incisal edge, middle third, and cervical third. The measurements were recorded before and after the finishing and polishing procedures. The initial thickness was 0.5 mm, and after finishing and polishing, the final thickness was approximately 0.4 mm, classifying the laminates as thin veneers. All adaptation procedures were performed following a strict protocol to maintain restoration integrity. Adjustments were minimal and conducted using fine-grit diamond burs (45 μm, KG Sorensen, São Paulo, Brazil) under copious water cooling (50 mL/min). The finishing and polishing sequence utilized the Exa Cerapol system in three steps: grey rubber for initial smoothing, pink for intermediate polishing, and white for final high-gloss polish. Each veneer underwent quality control inspection under 10× magnification using a dental loupe with LED illumination (EyeMag Pro S, Carl Zeiss, Germany) to detect any potential surface defects, chips, or crack lines. Any specimens showing visible defects were excluded from the study and replaced. This inspection protocol was performed both after the milling process and after any necessary adjustments to ensure the structural integrity of the veneers before cementation.

The cementation method was the same for both groups and in line with the manufacturer’s recommendations. It began with acid etching of the ceramic restorations with 10% hydrofluoric acid for two minutes for feldspar porcelain laminates and 5% for 20 s for lithium disilicate laminates. The acid was then washed for 30 s before the laminates were lightly air-dried. The next step involved the application of a silane agent (Monobond Plus, Ivoclar Vivadent, Schaan, Liechtenstein) to the inner surface of the ceramic piece for 60 s with an applicator brush, followed by drying with air. For the dental element, acid phosphoric 37% was used for 30 s, followed by washing and removing excess water with a light jet of air. This was followed by the application of two layers of universal adhesive (Adhese Universal, Ivoclar Vivadent, Schaan, Liechtenstein) for 10 s, followed by a light air jet and photoactivation for 10 s with LED-type light curing (Bluephase N, Ivoclar Vivadent, Schaan, Liechtenstein). Finally, the resin cement (Variolink Esthetic DC, Ivoclar Vivadent, Schaan, Liechtenstein) was applied to the inner surface of the thin ceramic laminate. The restoration was then adhered to the tooth with gentle digital pressure before any excess was removed. Next, the whole set was photopolymerized by applying the nozzle of the light-curing device to the center of the buccal face of the tooth for 60 s. The edges of the thin ceramic laminate were then covered with water-soluble glycerin to remove the air barrier before being polymerized for an additional 20 s. This cementation protocol was chosen based on each material’s microstructure. The different etching protocols (10% HF/2 min for feldspathic, 5% HF/20 sec for lithium disilicate) were selected to achieve optimal surface treatment while preventing excessive ceramic degradation. The dual-cure cement system ensures complete polymerization, while the glycerin barrier technique prevents oxygen inhibition layer formation, both crucial for long-term restoration stability.

Four specimens from each group were tested in a universal testing machine (EMIC, USP, São Paulo, Brazil) to determine the fracture load of thin ceramic laminates. The specimen alignment was standardized using custom-fabricated alignment jigs to ensure consistent positioning. Each specimen was embedded in transparent PVC tubes using acrylic resin, maintaining a precise 7° angle relative to the buccal face. This angle was verified using a digital angle gauge before testing. The mounting procedure was calibrated using reference marks on both the specimen holder and the testing machine to ensure reproducible positioning across all samples. The tubes containing the specimens were placed in the universal testing machine and subjected to a fracture load test (speed 0.5 mm/min). The load was applied to the incisal edge by means of a metal roller (length 15 mm, diameter 1.55 mm). The fracture load was recorded in Newtons (N), and the fractured specimens were stored for later fractographic analysis.

To minimize testing variables, specimens were carefully handled and monitored for potential errors such as misalignment, non-uniform load application, and load cell fluctuations.

**Figure 2:**
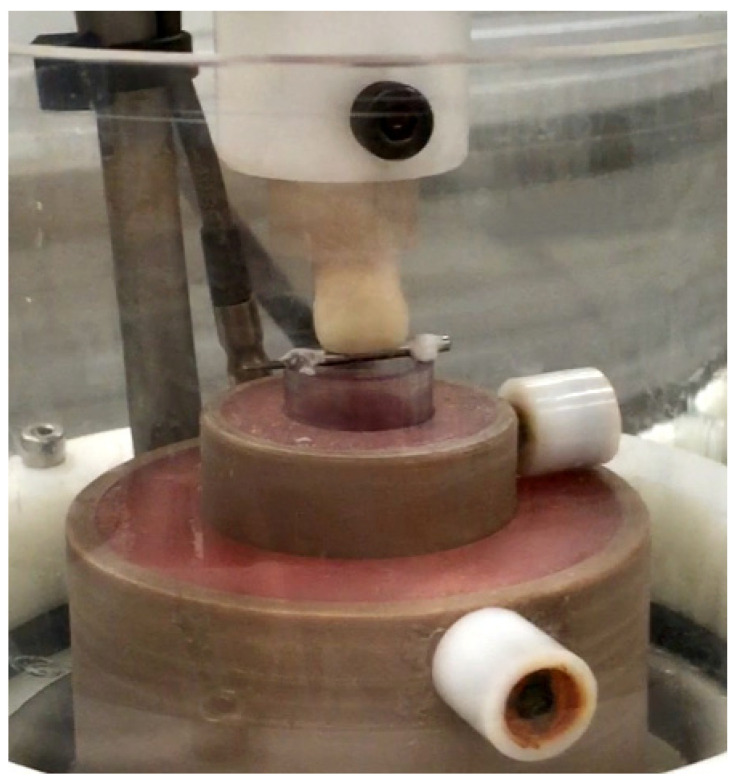
Placement of the specimens in the chewing machine simulator.

For the development of a methodology capable of determining the lifetime of thin ceramic laminates, one specimen from each group was tested using different parameters, including the type of antagonist (natural teeth or metal rollers), load level (20, 30 or 40 N) and masticatory cycle scheme (incision or sliding). Acrylic resin blocks served as a mounting base to secure the antagonists within the mechanical cycler and were positioned on the masticating simulator, as depicted in Figure 2.

The force parameters (20 N, 30 N, and 40 N) were selected based on established biomechanical studies of anterior tooth function, representing conservative loading (20 N for normal incising forces), typical anterior guidance forces (30 N for protrusive movements), and maximum physiological loading (40 N for parafunctional activities). A cycling frequency of 1.5 Hz was used to replicate the natural mastication rhythm. Two loading patterns were employed: an “incision” cycle simulating food-cutting mechanics and a “sliding” cycle replicating protrusive movements, ensuring evaluation under clinically relevant conditions. The specimens were subsequently subjected to cyclic fatigue on the chewing simulator (SD Mechatronik GmbH, Feldkirchen, Germany) until the failure of the thin ceramic laminate occurred. The force in the chewing simulator was produced by two pitch motors that allowed vertical and horizontal controlled computer movements between the specimen and the antagonist in each test chamber. Two types of masticatory cycles were used in this study. The first simulated how teeth cut off the food, called “incision”, and the second the movement of protrusion of the teeth during chewing, “sliding”. The application of force during the incision cycle consisted of a three-step motion, starting with a downward movement of 3 cm until the antagonist touched the incisal third of the palatal face of the thin ceramic laminate. After contact, the load was applied, and a protrusive movement simulating the sliding of the teeth during the masticatory function was performed. The last part of the cycle involved the return to the touch position in the incisal edge of thin ceramic laminate.

The application of forces during the sliding cycle was composed of a two-step motion, starting with the specimen and the antagonist tooth in MIC. With the onset of movement, the antagonist moved 3 mm in the direction of protrusion and returned to the starting point, always maintaining contact between the antagonist and the tooth. The chewing simulator parameters were changed for different specimens, and five different mechanical cycling conditions were tested, as described in Table 2. In condition 1, the specimens were tested with the natural tooth antagonist with a 40 N load using the incision cycle. In condition 2, the specimens were tested with natural teeth antagonists and a 30 N load using the incision cycle type. In condition 3, the antagonists used were also natural teeth with a load of 30 N, but the type of cycle performed was sliding. In condition 4, the specimens were tested with metal roller antagonists with a 30 N load on the sliding cycle. And in condition 5, the antagonists used were metal rollers with a load of 20 N in the sliding cycle.

The final frequency of all cycles performed was 1.5 Hz. During the tests, the specimens were immersed in distilled water at 37 °C and visually inspected for cracks or fractures. The number of cycles until the failure of the specimens was recorded. The macroscopic failure pattern of the thin ceramic laminates was analyzed via the naked eye, and the fracture surfaces were analyzed in a stereomicroscope (CCD, Olympus, Tokyo, Japan) to identify the failure modes.

### Statistical Analysis

For the fracture load testing, data normality was first verified using the Shapiro–Wilk test. The differences in fracture loads between the two ceramic materials (lithium disilicate and feldspathic porcelain) were analyzed using an independent *t*-test. To ensure the equal variance assumption of the *t*-test, Levene’s test was also conducted. For the fatigue testing, descriptive statistics were used to report the number of cycles until failure under different testing conditions. The failure modes were analyzed qualitatively through microscopic examination and reported as frequency distributions. All statistical analyses were performed using SPSS version 24 (IBM Corp., Armonk, NY, USA), with a significance level set at α = 0.05. 

## 3. Results

### 3.1. Fracture Load

Table 3 shows the mean values obtained during the fracture load test. The mean value obtained for lithium disilicate (431.8 ± 217.9 N) was statistically similar to that obtained for the feldspathic porcelain (454.4 ± 72.1 N). However, the standard deviation and coefficient of variation obtained for the lithium disilicate were higher than those obtained for the porcelain, indicating a higher spread of the fracture load data for the first. Specifically, the lithium disilicate group demonstrated notably higher data dispersion, with a standard deviation of 217.9 N and a coefficient of variation of 50.5%, compared to feldspathic porcelain’s standard deviation of 72.1 N and coefficient of variation of 15.9%. This increased variability in lithium disilicate’s fracture resistance may be attributed to factors such as the presence of internal defects, variations in crystal distribution, or processing-induced structural inconsistencies. From a clinical perspective, while both materials showed similar mean fracture resistance values exceeding normal masticatory forces (approximately 150–200 N), the higher variability in lithium disilicate suggests the need for careful quality control during fabrication and potential implications for long-term predictability in high-stress areas.

### 3.2. Stereomicroscopy and Scanning Electron Microscope

**Figure 3:**
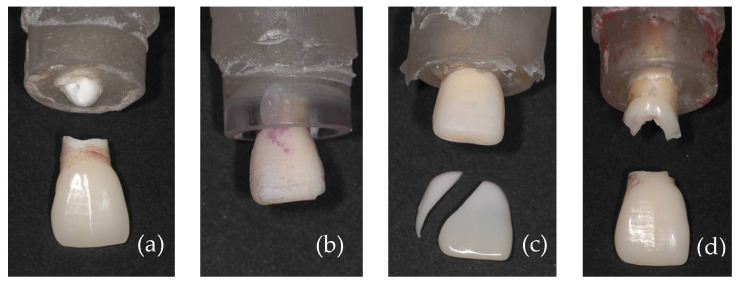
Placement of the specimens in the chewing machine simulator.

Figure 3 shows the overview images of lithium disilicate specimens after the fracture load test. In Figure 3a, specimen 1, it is possible to note that loading resulted in the fracture of the dental root; in Figure 3b, specimen 2, a small chipping of the incisal edge at the mesial side of the ceramic laminate veneer was observed; in Figure 3c, specimen 3, it is possible to observe a detachment of the ceramic laminate veneer with posterior fracture of the laminate; and in Figure 3d, specimen 4, the fracture line is at the level on the cervical part of the root.

**Figure 4:**
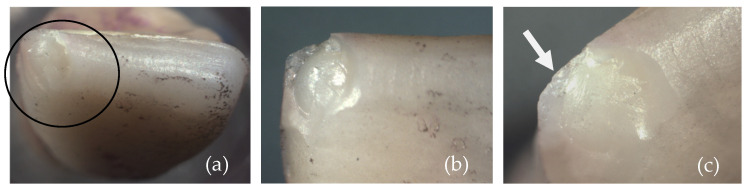
Images of the fracture surface of lithium disilicate specimen 2; (**a**) overview; (**b**) approximated view; and (**c**) possible source of failure.

**Figure 5:**
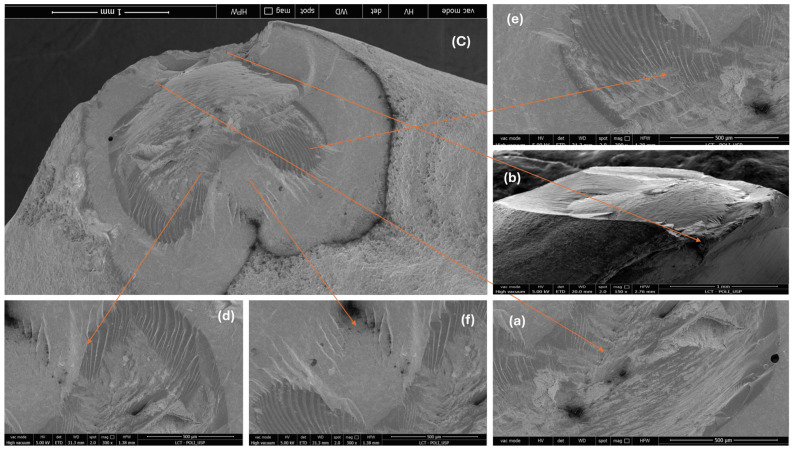
Images of the fracture surface of lithium disilicate specimen 2; (**a**–**f**).

Figure 4 and Figure 5 show images obtained via the stereomicroscope and scanning electron microscope, respectively. It is possible to observe the fracture patterns of lithium disilicate for specimen 2. The mesial side of the incisal edge shows a clear chip fracture with the origin at the point of contact with the indenter (palatal side).

**Figure 6:**
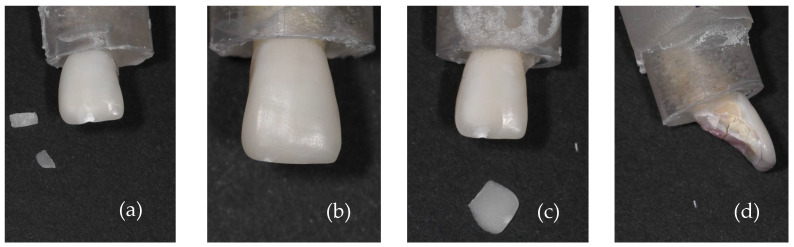
Images of fracture patterns of porcelain laminates; (**a**) specimen 1: fracture and chipping of the laminate veneer; (**b**) specimen 2: chipping of the laminate veneer; (**c**) specimen 3: chipping of the laminate veneer; and (**d**) specimen 4: fracture of the dental crown.

Figure 6 shows the overview images of the porcelain specimens after the fracture load test. In Figure 6a, specimen 1, it is possible to note the chipped incisal edge of the laminate veneer; in Figure 6b, specimen 2, a smaller chipping located at the incisal edge is observed; in Figure 6c, specimen 3, a large chip is observed involving the incisal edge and the buccal surface; and in Figure 6d, specimen 4, the tooth crown fractured, leaving the veneer intact.

**Figure 7:**
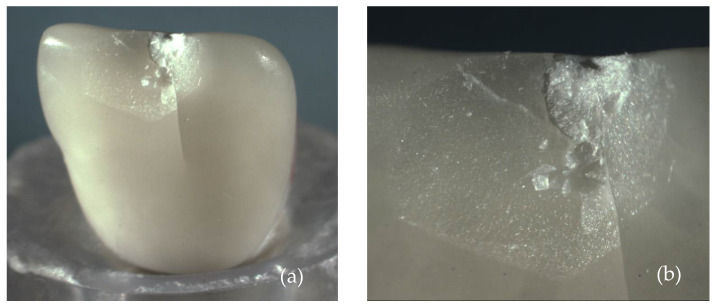
Images of the fracture surface of porcelain specimen 1; (**a**) overview; (**b**) approximated view and possible source of failure.

**Figure 8:**
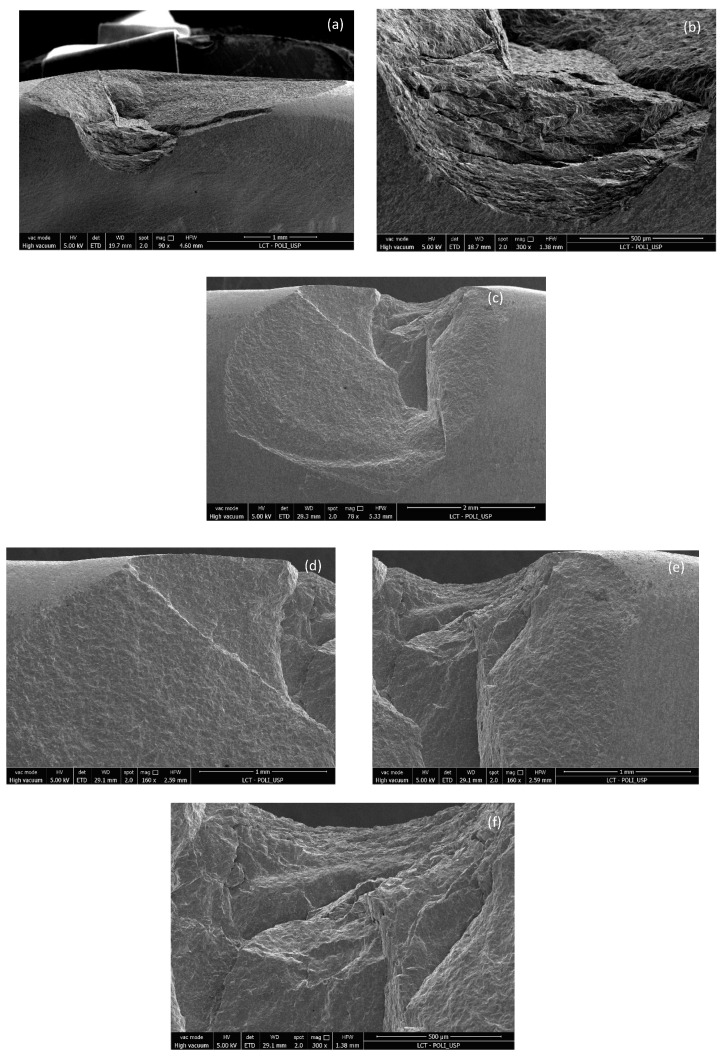
Images of the fracture surface of porcelain specimen 1; (**a**–**f**).

**Figure 9:**
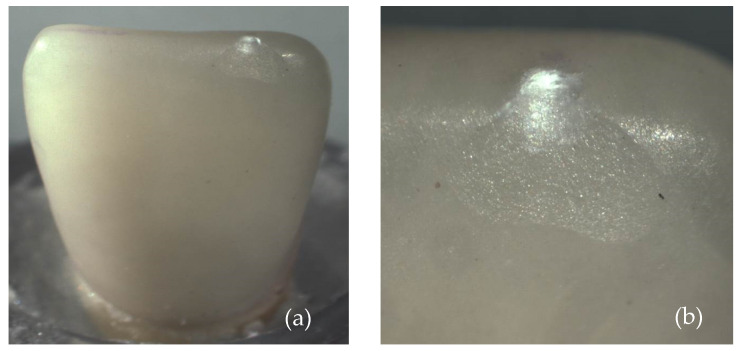
Images of the fracture surface of porcelain specimen 2; (**a**) overview; (**b**) approximated view and possible source of failure.

**Figure 10:**
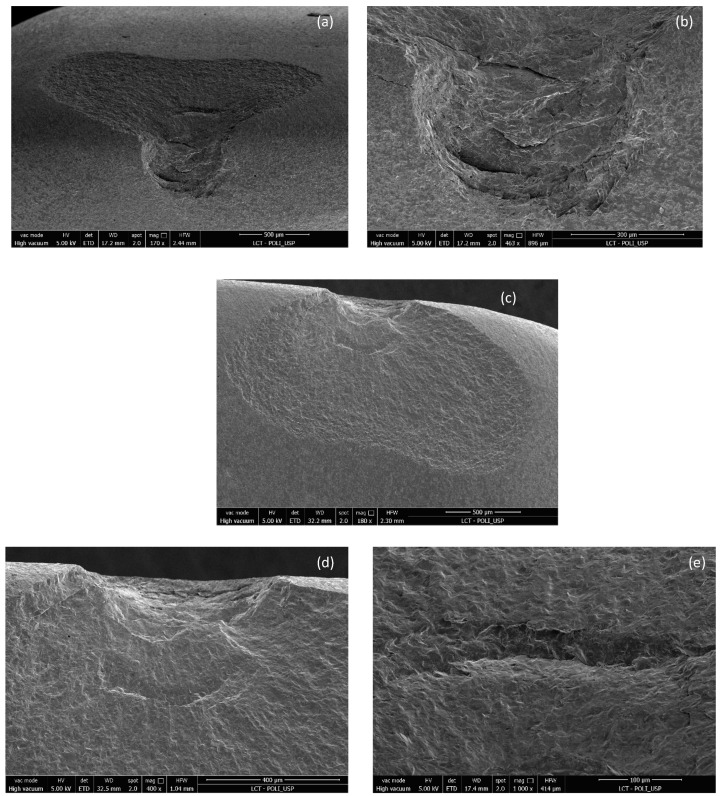
Images of the fracture surface of porcelain specimen 2; (**a**–**e**).

**Figure 11:**
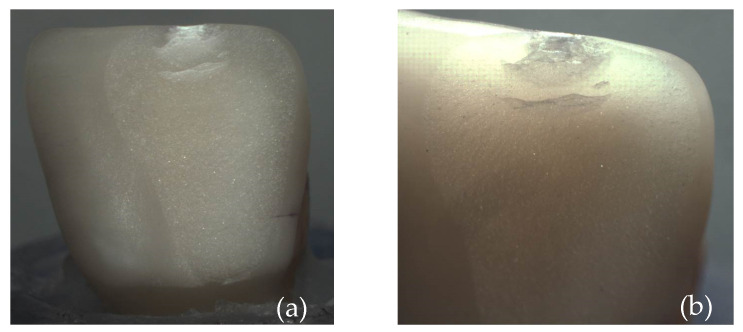
Images of the fracture surface of porcelain specimen 3; (**a**) overview; (**b**) approximated view and possible source of failure.

**Figure 12:**
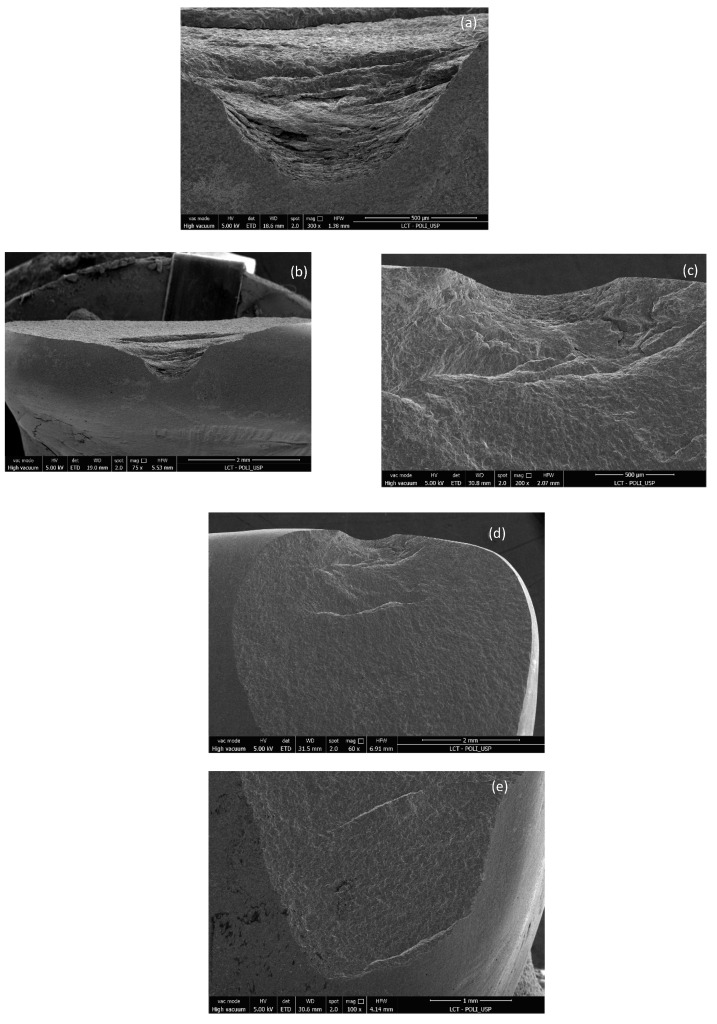
Images of the fracture surface of porcelain specimen 3; (**a**–**e**).

Figure 7, Figure 9 and Figure 11 show images obtained via the stereomicroscope, and Figure 8, Figure 10 and Figure 12 show images obtained via scanning electron microscope, in which it is possible to observe the fracture patterns of porcelain specimens used in the fracture load test. Figure 7 and Figure 8 show details of the fracture patterns of the porcelain for specimen 1. It is possible to observe the chipping of the incisal buccal edge with a crack propagating toward the cervical area of the veneer.

Figure 9 and Figure 10 show details of the small chipped area of the buccal incisal edge near the distal angle of the porcelain laminate veneer for specimen 2.

Figure 11 and Figure 12 show the chipped buccal edge extending to the cervical area of the porcelain laminate veneer specimen 3.

### 3.3. Lifetime Determination

Table 4 shows the results obtained in the chewing simulation tests for the different parameters tested. Five conditions were tested using different parameters during mechanical cycling. In conditions 1 and 2, the fracture of the antagonist occurred after 2534 cycles and 10,467 cycles, respectively. In condition 3, wear was observed on the incisal edge of the antagonist tooth, and the test was suspended after 103,772 cycles. In condition 4, the fracture of the thin ceramic laminate occurred with 5784 cycles and in condition 5, there was excessive wear on the metal roller, and with 536,818 cycles, the test was suspended.

**Figure 13:**
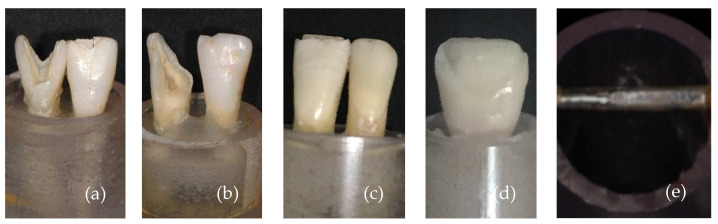
Images of failure patterns obtained in the chewing simulator; (**a**,**b**) fracture of the antagonist tooth; (**c**) wear of the antagonist tooth; (**d**) fracture of the laminate veneer; and (**e**) wear of the metal roller.

Figure 13 shows the failures observed during the chewing simulation tests. In Figure 13a,b, the antagonist tooth fractured during the tests corresponding to conditions 1 and 2, respectively. In Figure 13c, severe wear of the incisal edge of the antagonist tooth (condition 3) was observed. Figure 13d shows the fracture of the thin lithium disilicate laminate (condition 4). Figure 13e shows the excessive wear of the metal roller used in condition 5.

## 4. Discussion

This study found that the microstructure of the ceramic material did not affect the fracture load of the thin veneers tested, as there were no statistical differences between the mean fracture loads for feldspathic porcelain and lithium disilicate restorations. However, the failure modes of these materials differed significantly. Porcelain specimens primarily exhibited chipping at the incisal edge, while lithium disilicate veneers showed fractures involving both the dental root and larger areas of the veneers. Therefore, the hypothesis that both the fracture load and failure mode would be similar for both materials was only partially accepted. Regarding the second objective of the study, developing a cyclic fatigue methodology, the investigation encountered numerous challenges in modeling a mechanical cycling method capable of accurately simulating the complex stress distribution in anterior teeth. The novelty of this research lies in its comprehensive evaluation of failure patterns in minimally invasive ceramic veneers, particularly in establishing the relationship between material microstructure and failure modes under controlled laboratory conditions. This provides valuable insights for clinical decision-making in anterior restorations. Understanding masticatory load distribution is crucial when examining dental restoration performance. While healthy teeth distribute occlusal forces uniformly, endodontically treated teeth show altered load patterns due to structural changes, as demonstrated by Chieruzzi et al. [34]. Their findings on how post-treatments modify stress distribution within tooth structure are particularly relevant when evaluating the mechanical behavior of dental restorations. These factors highlight the potential risks of material selection in dental restorations, as the mechanical properties of the materials can directly influence the integrity of the underlying tooth structure.

Fracture load values have been used in many studies to characterize the mechanical behavior of ceramic restorations [35,36]. The strength of ceramic materials is limited by pre-existing defects and their fracture toughness [21,35]. Lithium disilicate ceramics typically have higher flexural strength than feldspathic porcelains [37]. However, this difference was not reflected in the current study’s findings. This discrepancy may be due to the experimental design, in which a thin veneer was produced and cemented to a prepared tooth, a situation in which stress distribution and defect types differ from those observed in simple bend bar tests for flexural strength [38].

Although the fracture load data for both materials were similar, the lithium disilicate specimens exhibited a higher coefficient of variation (51%) compared to porcelain specimens (16%), indicating greater variability in the size of defects that initiated failure [39,40]. One possible explanation for this variability is that lithium disilicate is more difficult to machine than feldspathic porcelain, potentially resulting in a wider distribution of defect sizes on the veneer surface [40,41,42].

Fractographic analysis revealed significant differences in the failure modes of the tested materials [43]. Porcelain veneers showed chips of varying sizes without root fractures, likely due to their relatively low fracture toughness compared to lithium disilicate [29,44,45]. The lower fracture toughness of dental porcelains is attributed to their high glass content (over 60%) and the presence of small amounts of leucite (up to 30%), which is insufficient to provide a fracture toughness greater than 1.0 MPa.m^1/2^. As a result, dental porcelains are more prone to slow crack growth and unstable crack propagation [46,47].

In contrast, the lithium disilicate veneers exhibited root fractures and debonding or fracture of the entire veneer. A possible explanation for the root fractures is that lithium disilicate can withstand higher loads before catastrophic failure [48] due to its higher fracture toughness of 3.5 MPa.m^1/2^ [18,49]. The material’s microstructure, composed of 70% lithium disilicate crystals embedded in a glassy matrix [50], contributes to its superior mechanical behavior compared to porcelain. Furthermore, the strong bond formed during the cementation process using phosphoric acid and silane allows the applied load to be efficiently transferred to the tooth structure, potentially exceeding the tooth’s ability to absorb energy before the lithium disilicate reaches its fracture toughness limit. The complex stress distribution along the entire tooth structure likely contributes to root fractures, as stresses concentrate in the cervical area of the tooth. Additionally, it is important to note that the teeth used in this study were devitalized, significantly reducing the resistance of the dental structure [40]. Understanding how these materials interact with tooth structure is essential for preventing failures.

The tests commonly used to determine the fracture load of ceramic materials are “crunch the crown” tests, in which an indenting tip is placed over the specimen and a load is applied until fracture occurs [51]. However, these tests do not fully replicate the clinical conditions ceramic crowns experience. Furthermore, fracture load data alone do not provide information about stress distribution in complex geometries, such as crowns, making it difficult to draw direct comparisons between studies [31,52]. It is important to differentiate between “crunch the crown” tests and fatigue studies that use specimens with geometries similar to clinical crowns, as the latter provide more relevant data by better simulating the clinical behavior of ceramic restorations [30,53].

Using simpler specimens, such as bars or disks, allows for standardized loading protocols with controlled loads, enabling comparisons across different studies. However, these data offer a conservative estimate of the material’s mechanical behavior [54] and do not account for the geometric factors necessary to simulate clinically relevant stress distributions. Additionally, the defect populations in bend bars differ significantly from those in thin veneers due to differences in how each is processed. To generate clinically relevant data, laboratory tests must simulate real-world behavior using complex specimens, such as crowns and veneers, and lower load levels applied cyclically [55,56].

This study aimed to develop a methodology involving cyclic loading of ceramic laminate veneers. The initial test design used an incisal masticatory cycle, with a 40 N load applied by a natural tooth antagonist. The tested specimen survived for 2534 cycles before the antagonist tooth fractured. Due to this early failure, the load was reduced to 30 N for the next test, which also employed the incisal pattern. In this test, the antagonist fractured after only 10,467 cycles, a result considered too rapid. To address this, the masticatory cycle was modified to minimize the impact on the antagonist.

The new design involved sliding the incisal edge of the opposing tooth onto the lingual surface of the ceramic veneer to better simulate the protrusive movement of incisors. In this test, the incisal edge of the antagonist tooth exhibited excessive wear after 103,772 cycles. As a result, a metal roller was introduced as the antagonist to withstand the forces of the experimental setup. This design can be applied in various fields of dental material testing and research, including evaluating the durability of ceramic materials, the effectiveness of different cementation techniques, and the performance of dental restorations under simulated masticatory conditions.

When using the metal antagonist, the veneer fractured after only 5784 cycles, suggesting that the mechanical cycling still generated high stress levels in the specimen. Therefore, the applied load was further reduced to 20 N, while maintaining the sliding movement and metal roller antagonist. Under these conditions, one specimen fractured after 536,818 cycles. However, the metal roller showed significant wear from the ceramic veneer.

Few studies have employed chewing simulators to evaluate thin ceramic laminate veneers, and test standardization is necessary for reliable comparison between studies [6,57,58,59]. The numerous variables present in the oral environment, and the difficulty in reproducing them in vitro, make this type of experiment challenging. While using a natural tooth antagonist would be ideal for testing dental enamel’s interaction with ceramic surfaces, the current study found it difficult to standardize antagonist teeth in terms of size and occlusal contact with the veneer. Consequently, a metal roller was deemed a better antagonist for in vitro studies, as it allowed for easier standardization.

In terms of masticatory movement, it would be ideal to expose specimens to both incisal cutting, which simulates food cutting, and sliding, which mimics the mandible’s protrusive movement, as both occur simultaneously in the oral cavity. However, the impact from incisal movement must be minimized to avoid excessive force on the veneer’s incisal edge. Controlling the applied load proved to be the most complex variable. Masticatory loads vary between individuals, and previous studies have reported differing results [60].

The clinical implications of our findings are significant for dental practice. The observation that feldspathic porcelain tends to exhibit localized chipping rather than catastrophic failure suggests it may be preferable in cases where preserving the underlying tooth structure is paramount. Conversely, lithium disilicate’s higher fracture toughness may make it more suitable for cases requiring maximum restoration durability, provided the risk of root fracture is carefully considered. It is important to acknowledge several limitations of the present study. The relatively small sample size used in this study may have affected the statistical power of our findings, although this number is consistent with previous research. Additionally, the study did not include thermal cycling as part of the aging process.

The findings highlight the critical role of the microstructure of ceramic materials and their mechanical behavior in clinical dental practice. Incorporating these considerations is crucial for mitigating the risk of tooth structure failures due to material selection and mechanical behavior. Further research should explore the implications of our findings on the development of more effective restoration methodologies and the potential for optimizing material selection based on specific clinical scenarios.

## 5. Conclusions

The microstructure of the ceramic material did not influence the fracture load of thin ceramic laminate veneers under static testing. However, the failure modes of the two materials differed significantly. Porcelain exhibited more chipping, while lithium disilicate showed a higher incidence of dental structure failures. The optimal combination of parameters for the mechanical cycling of ceramic laminate veneers could not be determined. The maximum number of cycles before fracture was 536,818, achieved using a metal roller antagonist with a 20 N load in a sliding mode without impact. These findings have important clinical implications for material selection, suggesting that clinicians should consider not only the material’s strength but also its potential impact on the underlying tooth structure when choosing between porcelain and lithium disilicate for laminate veneers. The different failure patterns observed between materials indicate that the preservation of dental structure might be influenced by material choice, which could affect long-term restoration success. Future studies should focus on the long-term clinical evaluation of failure modes, the investigation of alternative cycling parameters that better simulate intraoral conditions, and the development of standardized testing protocols for ceramic laminate veneers.

## Figures and Tables

**Table 1:** Experimental design.

Ceramic	n	Flexural Strenght	Chewing Simulation
Lithium disilicate (Ivoclar IPS e.max CAD)	9	4	5
Feldspathic porcelain (Vita Mark II)	9	4	5

**Table 2:** Mechanical cycling conditions tested.

Condition	Antagonist	Load	Cycle Type
Condition 1	Natural tooth	40 N	Incision
Condition 2	Natural tooth	30 N	Incision
Condition 3	Natural tooth	30 N	Sliding
Condition 4	Metal roller	30 N	Sliding
Condition 5	Metal roller	20 N	Sliding

**Table 3:** Mean values of fracture load in Newton with their respective standard deviation and coefficient of variation (in parentheses).

Material	Fracture Load (N)
Lithium disilicate (IPS e.max CAD)	431.8 ± 217.9 (51%)
Porcelain (Vita Mark II)	454.4 ± 72.1 (16%)

**Table 4:** Number of cycles until fracture and fracture pattern according to the parameters used in the chewing simulator.

Antagonist	Load (N)	Cycle Type	Number of Cycles to Fracture	Fracture Pattern
Natural tooth	40	Incision	2534	Fracture of the antagonist tooth
	30		10,467	Fracture of the antagonist tooth
		Sliding	103,772	Excessive wear of the antagonist tooth
Metal roller	30	Sliding	5784	Fracture of the thin ceramic laminate
	20		536,818	Excessive wear of the antagonist

## Data Availability

The data presented in this study are available on request from the corresponding author.
